# The Role of the Claustrum in Parkinson’s Disease and Vascular Parkinsonism: A Matter of Network?

**DOI:** 10.3390/life15020180

**Published:** 2025-01-26

**Authors:** Marialuisa Zedde, Rocco Quatrale, Gianni Cossu, Massimo Del Sette, Rosario Pascarella

**Affiliations:** 1 Neurology Unit, Stroke Unit, Azienda Unità Sanitaria Locale-IRCCS di Reggio Emilia, Viale Risorgimento 80, 42123 Reggio Emilia, Italy; 2 Dipartimento di Scienze Neurologiche, UOC di Neurologia—Ospedale dell’AngeloAULSS 3 Serenissima, 30174 Venice Mestre, Italy; rocco.quatrale@aulss3.veneto.it; 3 Neurology Unit, Dept of Neuroscience, ARNAS Brotzu, 09047 Cagliari, Italy; giovannicossu1@gmail.com; 4 Neurology Unit, IRCCS Ospedale Policlinico San Martino, 16132 Genoa, Italy; massimo.delsette@hsanmartino.it; 5 Neuroradiology Unit, Azienda Unità Sanitaria Locale-IRCCS di Reggio Emilia, Viale Risorgimento 80, 42123 Reggio Emilia, Italy; pascarella.rosario@ausl.re.it

**Keywords:** claustrum, middle cerebral artery, Parkinson’s disease, neurodegenerative, vascular parkinsonism, stroke, SVD, white matter hyperintensities

## Abstract

Background: The mechanisms underlying extrapyramidal disorders and their anatomical substrate have been extensively investigated. Recently, the role of the claustrum in Parkinson’s disease and other neurodegenerative conditions has been better detailed. The main aim of this review was to summarize the supporting evidence for the role of the claustrum in degenerative and vascular parkinsonism. Methods: The anatomy, biology, vascular supply, and connections of the claustrum in humans were identified and described, providing the substrate for the vascular involvement of the claustrum in large- and small-vessel disease. The vascular supply of the claustrum includes up to three different sources from a single artery, the middle cerebral artery, and it is known as territory with an intermediate hemodynamic risk. The connections of the claustrum make it a sensory integrator and a relevant point in several networks, from consciousness to movement planning. Conclusions: The claustrum is still an incompletely explained structure. However, recent description of its multiple connections indicate that it is involved in several diseases, including Parkinson’s disease. The evidence underlying its potential role in vascular parkinsonism is still scarce, but it might be a field warranting future investigations.

## 1. Introduction

The claustrum is a thin, gray matter structure that is located near the cortical surface of the insula, between the insula and putamen. It has extensive connections to multiple brain regions, including the prefrontal cortex (PFC), anterior cingulate cortex (ACC), entorhinal cortex (EC), hippocampus, amygdala, and insula. This network of connections enables the claustrum to play an important role in various cognitive functions. The claustrum is home to specialized neurons, such as Von Economo neurons (VENs) and position-sensitive cells, along with a dense population of claustral neurons, contributing to its involvement in attention, executive function, memory, language, and visuospatial abilities [1,2,3,4,5].

The role of the claustrum in human brain function remains enigmatic. Its small, complex shape and deep-seated location make it challenging to study, with few cases of isolated human lesions available for analysis [6,7]. Previous theories have linked the claustrum to consciousness [1,8], but recent research has shown its involvement in several processes. These include sleep regulation and slow-wave activity [9,10,11], saliency detection [12,13], and attentional load management [4,14,15].

The claustrum’s physiological role appears rooted in its selective inhibitory control over the cortex, enabling distributed and coordinated cortical activity—a mechanism potentially underpinning its various functions [3,16]. Furthermore, structural changes in the claustrum have been observed in several neurological and psychiatric conditions, including epilepsy [17], autism [1], encephalitis [18], schizophrenia [19], Parkinson’s disease [20], and prematurity-related disorders [21,22].

In Alzheimer’s disease (AD), the claustrum and its interconnected circuits undergo significant pathological changes. Amyloid plaques and neurofibrillary tangles, which are characteristic of AD, accumulate in the claustrum over the course of the disease. Specifically, plaques begin to form in the third phase of amyloid beta (Aβ) deposition, while neurofibrillary tangles appear in the claustrum at later stages of the disease [23,24]. Notably, AD patients with delusional symptoms show marked gray matter loss in the left claustrum, highlighting its vulnerability in this condition. Neuronal degeneration and synaptic damage, particularly in the anterior region of the claustrum, are considered key pathological features in AD [25,26].

The claustrum’s pathological involvement in Parkinson’s disease (PD) and dementia with Lewy bodies (DLB) highlights its potential role in non-motor symptoms, particularly dementia and visual hallucinations. Its extensive connections with cortical and subcortical regions implicated in these symptoms provide a plausible anatomical and functional basis for its contribution. Further research into the claustrum’s role in multisensory integration and network dynamics may shed light on its significance in these disorders, paving the way for targeted therapeutic approaches. In addition, the claustrum’s involvement in cognition, with its connections to the entorhinal cortex and amygdala (regions that are critical for memory and emotional processing), might contribute to cognitive impairment in AD and PD.

The main aim of this review was to describe the possible causative role of the claustrum in extrapyramidal manifestations, postulating how acute or chronic vascular damage may be associated with parkinsonism.

## 2. Anatomical Notes

The claustrum is a small, gray matter structure primarily composed of glutamatergic excitatory neurons and GABAergic interneurons [27]. In humans, it is located between the external and extreme capsules, beneath the insular cortex of the brain [16], but in other species, such as rodents, there is no capsula extrema [6]. While the claustrum is found in all mammals [28], its exact shape, position, and connections differ across species [27].

During development, the cells of the primordial claustrum originate from the radial progenitor domains of the lateral pallium, forming in parallel with the subplate cells of the dorsal pallium. This process involves tangential migration between the two areas, making the claustrum one of the earliest-formed structures in the forebrain [27,28,29,30]. Despite accounting for only 0.25% of the cortex’s total volume [28], the claustrum is considered the most densely interconnected forebrain region by volume [31]. This dense network of connections makes the claustrum a particularly intriguing structure for the pathophysiology of several degenerative and acquired diseases.

Morphological differences and cortical connections divide the claustrum into two distinct regions:

- Dorsal (insular) claustrum: situated above the rhinal fissure, medial to the insular cortex, this region has been extensively described in the literature [32].
- Ventral (piriform) claustrum or endopiriform nucleus: Located below the rhinal fissure, medial to the piriform cortex, this part is sometimes termed the endopiriform nucleus. Paxinos and Watson [33], after studying rats’ cortexes, subdivided it into the dorsal (DEN) and ventral (VEN) endopiriform nuclei, with the DEN localized rostrally to the pre-endopiriform nucleus [34].

The modern classification often distinguishes the dorsal claustrum (CL) from the ventral endopiriform nucleus (END), reflecting their anatomical and functional differences. Based on the expression of transcription factors during development, the claustrum and amygdaloid nuclei are thought to originate from the lateral and ventral pallium. The dorsal or insular claustrum is classified as a derivative of the lateral pallium, while the ventral claustrum, also known as the endopiriform nucleus, is considered a derivative of the ventral pallium. Together, the CL, END, and pallial amygdala form a unified structural and developmental entity referred to as the claustro-amygdaloid complex [35,36].

The shape of the claustrum varies across species, but generally scales with the neocortical volume. A volumetric analysis [28] revealed a proportional relationship between the log-volume of the claustrum and the log-volume of the cerebral hemisphere. The claustrum-to-cerebral hemisphere volume ratio ranges from 0.99% in mice to 0.24% in humans. Across all the examined species, the dorsal claustrum consistently occupies a larger proportion of the total claustral volume, accounting for 57.0–74.6%.

The claustrum is generally agreed to consist of two distinct neuronal subpopulations:

- Projecting neurons, which are characterized by medium-sized to large polymorphic cell bodies and spiny dendrites.
- Interneurons, which are identified by medium-sized to small round or oval cell bodies and aspiny dendrites [37].

The anatomy of the human claustrum can be studied through axial, coronal, and sagittal slices of post-mortem brains, revealing its topology and macroscopic structure. These studies divide the claustrum into two parts (Table 1).

Using Klingler’s microsurgical fiber-dissection technique, the white matter architecture can be examined [38]. After removing the frontal, parietal, and temporal operculae, the cortical surface of the insula is exposed. The removal of the gray matter of the insular gyri reveals the extreme capsule and the claustrum. The extreme capsule contains (I) a ventral component, with lateral fibers of the uncinate and inferior occipitofrontal fascicles, and (II) a thinner dorsal component, with short association fibers connecting the insular gyri to adjacent frontal, parietal, and temporal operculae. Dorsal external capsule fibers converge and merge with the dorsal claustrum gray matter, forming a spoke-and-wheel pattern. These fiber dissection studies challenge the traditional static view of the external capsule, which was once thought of as merely a group of fibers beneath the claustrum, instead proposing a dynamic view. The external capsule can now be seen as part of the external capsule found in the claustro-cortical projection system that interconnects the claustrum with multiple cortical regions [38,39].

**Figure 1:**
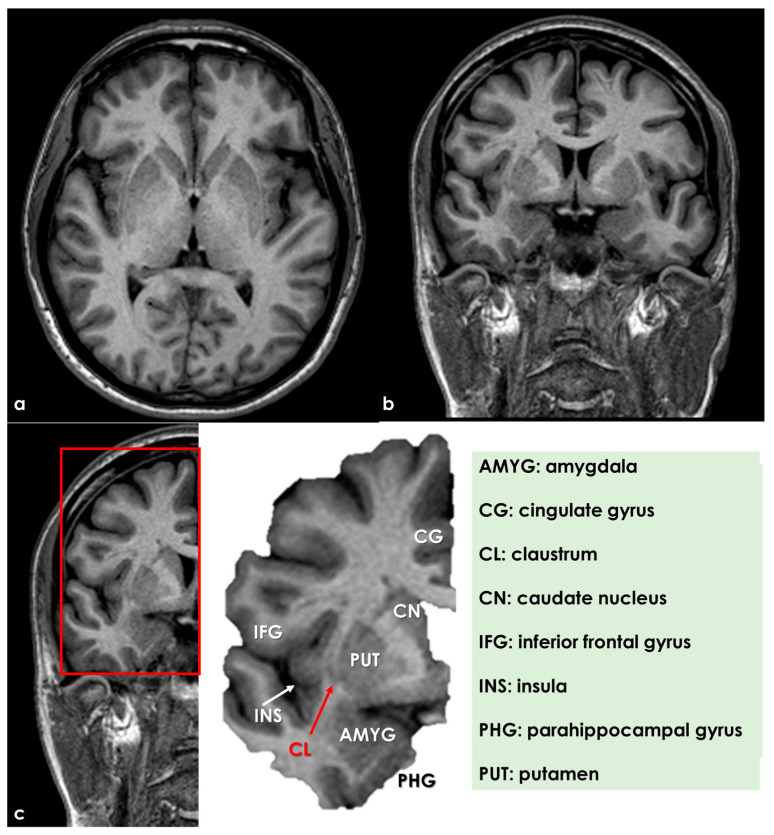
Brain magnetic resonance imaging (MRI) showing the localization and neighbor structures of the human claustrum. Panel (**a**,**b**) show axial and coronal T1W MRI, respectively, at the level of the insula in a normal subject. Panel (**c**) shows a detail of the right hemisphere in the coronal plane and a further magnification of the brain structures behind the claustrum (right portion of the panel).

**Figure 2:**
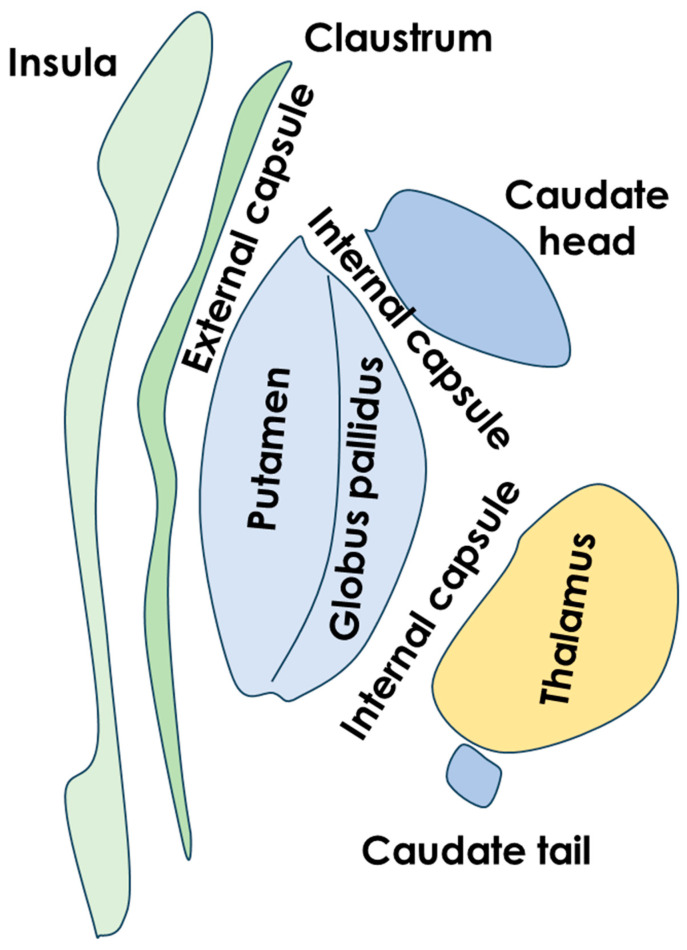
Schematic drawing of the basal ganglia and claustrum in axial view.

An example of the claustrum position and the surrounding structures is illustrated in Figure 1 and Figure 2.

Several studies involving white matter fiber dissection have extensively described the claustro-cortical system [38,39,40]. Once the sulci and gyri of the insula are removed, particularly around the apex and limen insulae, the extreme capsule becomes visible. They both consist of several association fibers that connect the frontal, parietal, and temporal opercula to the short and long insular gyri [41]. Upon excising the thin white matter layers that form the extreme capsule, the gray matter fibers of the dorsal claustrum are exposed. The dorsal claustrum lies in proximity to the posteroinferior aspect of the insula and is closely associated with the external capsule, located deep to the claustrum. During dissection, both structures are visualized, creating a classic cogwheel arrangement of fibers. When the anterior portion of the extreme capsule is removed, the ventral claustrum is revealed. In the anterior part, near the insular isthmus, the uncinate fasciculus (UF) becomes visible, while the inferior fronto-occipital fasciculus (IFOF) remains associated with the posterior two-thirds of the insular isthmus. The medial fibers of the ventral claustrum form part of the external capsule. Continuing the dissection from lateral to medial, as the ventral claustrum is removed, the external capsule fibers become clearly distinguishable. These fibers, especially in the medial and superior regions, merge with the fibers of the corona radiata, located deep in the superior longitudinal fasciculus (SLF). The dorsal part of the external capsule is situated beneath the dorsal claustrum. Differentiating the dorsal claustrum from the external capsule is challenging, as removing fibers from the dorsal external capsule also removes parts of the dorsal claustrum, and vice versa. At this level, the fibers of both structures are tightly intertwined; in fact, the claustrum and the external capsule are highly connected. The dorsal part of the claustrum is compact; in contrast, its ventral part is made of small islands of gray matter intermingled with the fibers of the trunk of the IFOF and UF. The external capsule, which is medial to the claustrum, is also subdivided into dorsal and ventral parts: the dorsal external capsule contains radiate claustro-cortical fibers, whereas the trunk of the UF and IFOF forms the ventral external capsule.

## 3. Vascular Supply

The claustrum receives its blood supply from three distinct arterial groups originating from the middle cerebral artery (MCA). The first group of arteries enters through the limen of the insula, primarily supplying the anterior and ventral regions of the claustrum. The second group traverses the insular cortex, mostly vascularizing the dorsal part of the claustrum. The third group, stemming from lateral striated arteries, infrequently provides blood to the claustrum, a phenomenon observed predominantly in adulthood. The primary sources of the blood supply to the claustrum are the arteries passing through the limen and cortex of the insula, as they predominantly contribute to its vascularization. In contrast, the arteries originating from the lateral striated arteries are considered secondary sources, playing a minor and infrequent role in the blood supply to the claustrum.

**Figure 3:**
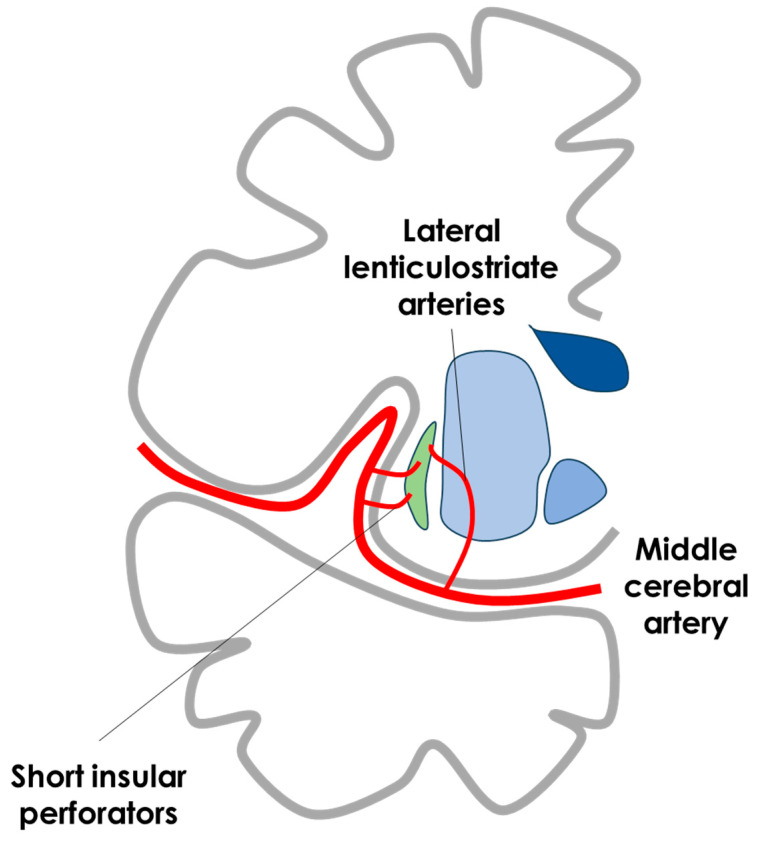
Schematic drawing of the arterial supply of the claustrum in coronal view with the same color code as in Figure 2.

The M1 MCA perforators, the anterolateral central (or anterolateral lenticulostriate) arteries, mainly originate from the pre-bifurcation segment of M1 and ascend to reach the anterior perforated space. Depending on their origin, they are subdivided into lateral, medial, and sometimes intermediate groups. The lateral and intermediate groups run through the putamen to vascularize the head and body of the caudate nucleus and the superior part of the internal capsule. The medial group vascularizes part of the globus pallidus, the anterior limb of the internal capsule, and the head of the caudate [42]. After the sharp angle, or genu, between the M1-sphenoidal and M2-insular segments of the MCA, the trunks of the MCA approximately divide in a mean of eight stem arteries [38,39,40,41,43,44,45,46] fanning at the surface of the insula [44]. These stem arteries divide into two or more cortical arteries that reach the circular sulcus of the insula, which is considered to be the distal limit of the M2 insular segment of the MCA [44]. Most insular arteries are short [47] and only supply the insular cortex and underlying extreme capsule; 10% are medium-sized and also vascularize the claustrum and external capsule; and 3–5% are long and reach deeper structures, such as the corona radiata [47]. In each hemisphere, an average of 96 insular-perforating arteries supply the insula. The majority of the insular arteries (75–104) arise from the M2 segment. In 55% of the hemispheres, the M1 segment gives off 1–6 insular arteries. In 25% of the hemispheres, the M3 segment branches off 1–2 insular-perforating arteries [44,47,48,49]. The middle-perforating arteries (MPr) constitute approximately 10% of the insular-perforating arteries. In addition, the same area of the short Pr supplies the claustrum and the external capsule. The number of SPr or MPr arteries with a size of 0.1–0.2 mm lying on the surface of the insula is 114. A schematic drawing of the arterial supply of the claustrum is illustrated in Figure 3.

The vascular supply of the claustrum is probably related to its ontogenetic similarities with the insular cortex. In fact, an additional function ascribed to the claustrum involves its role in salience processing. This attribution is supported not only by its anatomical proximity to the insula, but also by their shared developmental origins. Using immunohistochemistry techniques on human claustrum samples from the insular and temporal subregions, Pirone et al. [50] demonstrated that the claustrum shares its ontogeny with the insular cortex, but not with the putamen.

## 4. Claustrum Connections

The mammalian claustrum is a subcortical telencephalic structure characterized by diverse neuronal projections and interneurons [51,52,53,54]. Its anatomical proximity to surrounding structures, such as the insula and putamen, complicates the differentiation of neuronal circuits. This challenge arises from overlapping functional activities and the limitations of current evaluation methods, such as functional MRI (fMRI). Establishing a direct connection between neural signals and specific substrates remains difficult, as local stimulation may inadvertently activate nearby regions [53,54]. However, methods like a small region confound correction (SRCC) offer an improved accuracy in distinguishing claustral neural circuits [54]. Despite these advancements, the structural and functional dynamics of the claustrum require further exploration to overcome the current limitations.

Recent neuroanatomical studies indicate that the claustrum maintains an extensive network of connections with subcortical regions, including the hippocampus, thalamus, putamen, and basal nuclei, as well as cortical areas such as the temporal, occipital, and sensory lobes [38,55,56,57,58]. It remains unclear whether any cortical regions lack connections to the claustrum, despite it being one of the brain’s most densely connected structures per unit volume [55,56,59,60].

Tractography studies have identified two major projection pathways: the dorsal tract, which connects the claustrum to sensory and motor regions, and the ventral tract, which links it to auditory and olfactory regions [61]. Other research suggests four primary fiber tracts—anterior, posterior, upper, and lateral—connecting the claustrum to cortical areas such as the prefrontal cortex, visual regions, sensorimotor regions, and the auditory cortex, respectively [55]. The medial tract links the claustrum to basal ganglia structures, including the caudate nucleus, putamen, and globus pallidus, although the existence of such connections is debated [62,63]. Interhemispheric connectivity has also been observed through cortico-claustral and inter-claustral fibers [64].

**Figure 4:**
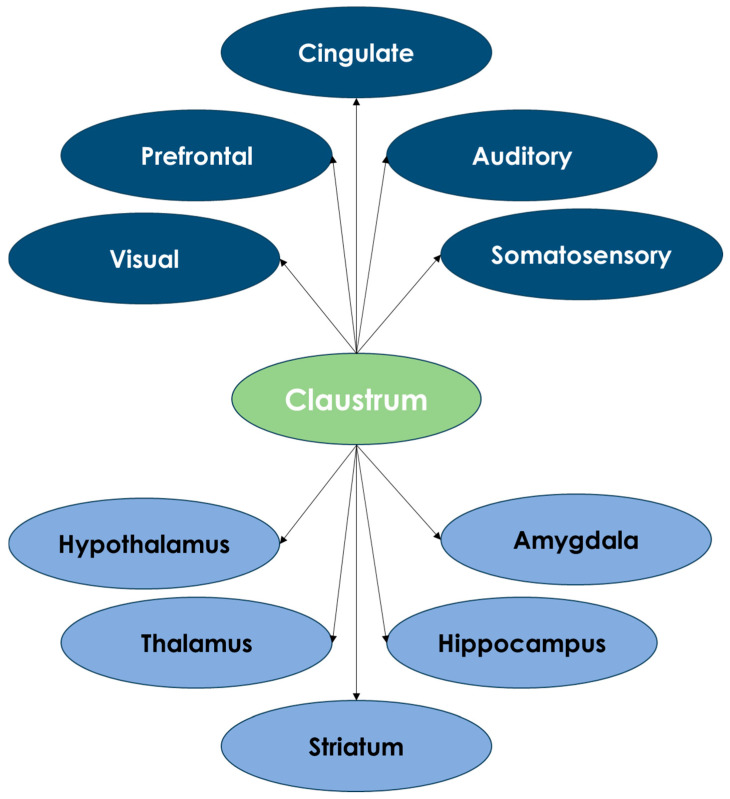
Schematic drawing of claustrum connections (cortical connections in blue and subcortical connections in light blue).

Claustral circuits demonstrate a strong affinity for the frontal cortex, including the anterior cingulate gyrus, prelimbic region, and medial prefrontal cortex, while showing limited connections to primary sensorimotor regions [54]. These circuits link the anterior cingulate gyrus to the visual and parietal cortices, enabling inhibitory processes critical for information processing and transmission [65]. This connectivity supports the claustrum’s role in spatial–temporal coordination across cortical areas [65]. Contralateral cortico-claustral projections are notably denser than ipsilateral ones [66]. The claustrum also receives input from subcortical structures, including the mediodorsal thalamus, basolateral amygdala, and hippocampus, and exhibits intraclaustral connectivity along the rostro-caudal axis. A schematic drawing of claustrum connections is illustrated in Figure 4.

Functionally, the claustrum fosters cognitive control by connecting with the prefrontal cortex, anterior cingulate gyrus, and secondary visual cortex. Cognitive tasks often involve the activation and deactivation of specific cortical regions through claustral circuits, independent of sensory–motor processing [54]. The claustrum is certainly involved in processes ranging from salience detection to multisensory integration for perceptual binding. Madden et al. [67] recently proposed a novel functional model claiming that frontal cortices direct the claustrum to flexibly instantiate cortical networks to subserve cognitive control.

## 5. The Claustrum and Parkinson’s Disease

The involvement of the claustrum in cortico-basal ganglia circuitry has been demonstrated in animal studies, including non-human primates [68]. In fact, the claustrum is an ancient telencephalic subcortical structure characterized by extensive reciprocal connections with much of the cortex, as well as inputs from the thalamus, amygdala, and hippocampus. It plays a general role in modulating cortical excitability and is implicated in a range of cognitive and motor functions, including sensory integration and perceptual binding, salience-guided attention, top-down executive processes, and the regulation of brain states, such as sleep and interhemispheric integration. In the macaque brain [68], claustral connectivity has a rough topographic organization. Notably, specific claustral zones project to both distinct striatal territories and associated cortical areas, which themselves contribute to the same striatal networks. These findings reveal additional layers of complexity in basal ganglia information processing for motor and non-motor functions, highlighting the claustrum’s influence on cortical functional domains and cortico-basal ganglia circuits.

PD is classically characterized by the degeneration of dopaminergic neurons in the substantia nigra, pars compacta (SNc). Clinical symptoms typically appear after the depletion of 80–85% of striatal dopamine and the loss of 50–70% of SNc neurons. While SNc neuron loss is essential for the pathological confirmation of PD, extranigral lesions affect various subcortical systems, leading to complex neurotransmitter dysfunctions and contributing to the disease’s clinical heterogeneity [69]. Beyond dopamine depletion in the striatum, a significant reduction in noradrenaline (from the locus coeruleus), serotonin (from the raphe nuclei), and acetylcholine (from the nucleus basalis of Meynert) occur due to degeneration in these regions [70,71,72]. Despite this widespread pathology, PD does not usually present with substantial brain atrophy, as neuronal loss is restricted to specific populations [73,74]. A hallmark of PD pathology is the presence of neuronal intracytoplasmic inclusions known as Lewy bodies (LBs) and neuronal process inclusions called Lewy neurites (LNs), both primarily composed of misfolded α-synuclein (αSyn). Mutations in the αSyn gene have been implicated in PD, and its aggregation underpins the molecular pathology of synucleinopathies, a group of disorders that includes multiple system atrophy (MSA), dementia with Lewy bodies (DLB), and others, with PD being the most common [75,76]. Although SNc degeneration and LB/LN presence in brainstem regions such as the locus coeruleus are diagnostic for PD, the disease affects multiple brain and peripheral regions. These include the olfactory bulb, the spinal cord, the dorsal motor nucleus of the vagus nerve, the pedunculopontine nucleus, the thalamic nuclei, the amygdala, the claustrum, the hippocampus, and various cortical regions, as well as autonomic nerves [77,78]. This widespread pathology likely accounts for PD’s non-motor symptoms.

The progression of αSyn pathology in PD is hypothesized to follow a predictable pattern, as outlined in Braak’s six-stage model [79]. The pathology begins in the dorsal motor nucleus of the vagus nerve and olfactory bulb (stage 1) and advances rostrally through the brainstem (stage 2), midbrain (stage 3), basal forebrain, and mesocortex (stage 4), before spreading to the neocortex (stages 5–6). While influential, this model has faced criticism regarding its methodology and clinical relevance [80]. Notably, αSyn pathology is frequently accompanied by AD-related changes, including amyloid plaques and neurofibrillary tangles, which may contribute to cognitive impairment in PD [69]. Molecular interactions between αSyn, tau, and amyloid-beta proteins have been demonstrated, highlighting shared mechanisms across neurodegenerative disorders [81,82].

The investigation of the claustral pathology in PD has been notably limited, with most studies focusing on other brain regions and often obscuring rather than elucidating the role of the claustrum. However, recent advances in research techniques have shown that the claustrum indeed undergoes pathological changes in PD [83,84,85]. One primary observation is the presence of Lewy pathology, including LBs and LNs, in the claustrum. The morphology of LBs in the claustrum aligns with the cortical type, characterized by less prominent features compared to the classical LBs observed in the brainstem. Typically, LBs are spherical inclusions ranging from 5 to 25 μm in diameter. Classical LBs exhibit a dark eosinophilic center surrounded by a pale halo when stained with hematoxylin and eosin. In contrast, cortical LBs in the claustrum, amygdala, and cortex appear less distinct. Immunohistochemistry using αSyn has become the standard for identifying LBs and LNs, revealing diverse αSyn inclusions. These include irregularly shaped deposits, particulate inclusions, and compact spherical inclusions resembling classical LBs. Additionally, extracellular αSyn deposits, likely remnants of neuronal intracytoplasmic inclusions, are often found in the claustrum following neuronal death. LNs in the claustrum exhibit various morphologies, including segmental and continuous serpentine patterns and pearl-like structures. These features are also present in other brain regions, such as the hippocampal CA2 sector, nucleus basalis of Meynert (NBM), amygdala, and lower brainstem structures [69]. A recent clinico-pathological study identified αSyn lesions in the claustrum in 75% of PD cases without dementia and 100% of cases with PD dementia (PDD) or DLB [69]. Astrocytic αSyn immunoreactive inclusions have also been identified in the claustrum of PD patients, but not in controls, highlighting a unique pathological feature [83]. In one reported case, a patient with clinical PD and neuropathological features of early MSA exhibited abundant αSyn-positive glial cytoplasmic inclusions (GCIs) in the claustrum [86]. GCIs are hallmark features of MSA, a progressive neurodegenerative disorder characterized by parkinsonism, ataxia, pyramidal signs, and autonomic dysfunction [86,87]. Other proteinopathies, such as tau pathology and amyloid-β (Aβ) deposits, have also been studied in the claustrum. Tau pathology, including neurofibrillary tangles (NFTs) and neuropil threads (NTs), is minimal in PD patients, irrespective of their dementia status [69]. However, Aβ deposits have been observed, with 25% of PD, 58% of PDD, and 100% of DLB cases exhibiting a claustral Aβ pathology [69].

A limited, but growing, body of research underscores the pathological involvement of the claustrum in PD. Observations include αSyn-immunoreactive inclusions, astrocytic changes, and protein deposits such as Aβ, suggesting that the claustrum may play a role in clinical manifestations of PD. Further investigation into the claustrum’s pathological contributions could provide new insights into the disease’s progression and symptoms. From a clinical point of view, non-motor symptoms associated with PD, particularly dementia and visual hallucinations, may be related to claustral pathology. PD has traditionally been characterized by its motor symptoms; however, increasing attention has been directed toward its non-motor symptoms, which significantly impair quality of life. Among these, dementia and visual hallucinations are particularly prevalent and impactful. Dementia develops in 48–78% of patients over the course of the disease [88], while visual hallucinations affect up to 60% of individuals with PD [89]. These symptoms are not unique to PD, but are also core features of DLB, a related neurodegenerative disorder recognized as the second most common cause of degenerative dementia after AD [90,91]. DLB is characterized by progressive cognitive decline, parkinsonism, fluctuating cognition, and well-formed visual hallucinations. Both PD and DLB share overlapping pathological hallmarks, including the presence of LBs and LNs, not only in the substantia nigra, but also in the cortical and subcortical regions, which likely underpins the spectrum of motor and non-motor symptoms [85]. Notably, the burden of αSyn pathology increases progressively from PD to PDD and is most pronounced in DLB. Similar trends have been observed for amyloid-beta (Aβ) deposition, while tau pathology appears negligible in these disorders. A study by Kalaitzakis and colleagues [85] investigated the αSyn and Aβ pathology in the claustrum of 39 cases (20 PD, 12 PDD, and 7 DLB). They observed significant differences in the αSyn burden between demented and non-demented PD cases, with the highest levels of pathology in the DLB cases. Despite these findings, there was no significant association between claustral pathology and the presence of visual hallucinations in this cohort.

Further evidence for the involvement of the claustrum in visual hallucinations comes from a study by Yamamoto et al. [92], which examined 20 DLB cases. The researchers noted an extensive LB and LN pathology in the claustrum, particularly in areas functionally connected to visual processing regions such as Brodmann areas 18 and 19, the insula, and the temporal cortex. These findings suggest that the claustrum may participate in visuo-claustral pathways, whose disruption could contribute to visual hallucinations. Clinical observations also support this hypothesis. For example, Ishii et al. [18] described a young patient with mumps encephalitis who experienced visual hallucinations and had bilateral symmetric lesions in the claustrum. This case highlights the potential of claustral damage to interfere with visual perception and cognitive integration.

The precise physiological role of the claustrum remains elusive, but evidence suggests that it acts as a “neural integrator,” binding multisensory and cognitive information across cortical and subcortical networks [7]. Its extensive reciprocal connections with regions implicated in visual, emotional, and memory processing—including the visual cortex [93,94], the amygdala [95], and the hippocampus [96]—position the claustrum as a key node in complex neural circuits. The pathological involvement of the claustrum in PD and DLB may, therefore, have far-reaching implications. While the claustrum may not serve as a primary epicenter for dementia or hallucinations, its role in binding and processing neural information suggests that its dysfunction could amplify disruptions in larger cortical–subcortical networks. This hypothesis aligns with studies demonstrating the claustrum’s involvement in higher-order cognitive functions, including memory, emotional processing, and behavioral regulation [97,98]. In the context of PD and DLB, dementia and visual hallucinations likely arise from widespread network dysfunction rather than isolated structural damage. The claustral pathology, while significant, is just one aspect of this broader dysregulation. For instance, visual hallucinations in DLB have been linked to LB pathology in the amygdala and parahippocampal cortex [99], while cognitive decline has been associated with αSyn and Aβ deposition in cortical and limbic regions [100], but also directly in the claustrum [85]. The findings from these studies underscore the importance of a network-based understanding of neurodegenerative diseases. The claustrum’s involvement in PD and DLB pathology is likely contributory rather than causative, reflecting its position within a complex web of neural circuits that underpin cognition, perception, and behavior.

Non-motor symptoms in PD extend beyond cognitive and sensory deficits, encompassing impulse-control disorders, sleep disturbances, and depression. Emerging evidence implicates the claustrum in these phenomena, suggesting that its pathology may contribute to a variety of behavioral and affective disorders observed in PD. In addition, impulse-control disorders in PD are characterized by an inability to resist urges or behaviors that can be harmful to oneself or others. Common manifestations include pathological gambling and hypersexuality, both of which are linked to dopaminergic dysregulation [101]. A positron emission tomography (PET) study [102] demonstrated heightened activity in the claustrum in healthy males exposed to sexual stimuli. This suggests that the claustrum plays a role in mediating pleasure and reward. In PD patients, pathological changes in the claustrum could lead to disinhibition, contributing to these maladaptive behaviors.

Sleep-related problems are a prevalent and debilitating non-motor symptom of PD, affecting both patients and their caregivers [103]. Périco et al. [104] investigated the relationship between cerebral blood flow and insomnia severity in patients with major depressive disorder. They found an inverse correlation between insomnia severity and blood flow in the claustrum, insula, and anterior cingulate cortex. These findings align with the claustrum’s known connections to sensory cortical regions and its afferents from the hypothalamus, thalamus, and locus coeruleus, regions critical for arousal and sensory processing. Claustral dysfunction in PD may disrupt this arousal–sensory integration, exacerbating sleep disturbances.

Depression is one of the most common psychiatric symptoms in PD, significantly reducing the quality of life [105]. A PET imaging study [106] investigated the cerebral metabolism in patients with unipolar and bipolar depression, revealing a correlation between psychomotor retardation, anhedonia, and reduced metabolism in the right insula, claustrum, anteroventral caudate/putamen, and temporal cortex. These findings suggest that the claustrum is involved in mood regulation and reward processing. In PD, pathological changes in this structure may contribute to depressive symptoms, particularly those characterized by low motivation and diminished pleasure.

This evidence underscores the claustrum’s involvement in several non-motor symptoms of PD, including impulse-control disorders, sleep disturbances, and depression. While the precise mechanisms remain unclear, the claustrum’s extensive connectivity with the cortical and subcortical regions implicated in emotion, reward, arousal, and sensory processing positions it as a key player in these phenomena. Further research is needed to elucidate the role of the claustral pathology in PD. No difference in the claustrum volume was found in AD or PD, but a significant change was found in the connections of the left claustrum with the sensorimotor and cingulate cortex in PD [107]. Clinico-pathological studies could clarify the extent to which claustral dysfunction contributes to these non-motor symptoms, offering potential targets for therapeutic intervention. The main pathological changes in the claustrum in PD are summarized in Table 2.

## 6. Claustrum Involvement in Vascular Parkinsonism

The arterial supply of the claustrum region is highly variable, with a main contribution from insular branches and a less constant contribution from lateral lenticulostriate arteries. This variability makes it a vulnerable territory in some circumstances. In fact, the area encompassing the external capsule, claustrum, and extreme capsule is supplied by the same two types of vessels found in the U-fiber region, entering through the insular cortex, i.e., the terminal branches of the longest cortical arterioles (Duvernoy type 5) and the early branches of long medullary arteries and arterioles (Duvernoy type 6). These vessels are considered intermediate in length. For any given region of U fibers, these two types of afferent vessels typically originate from different locations on the brain surface, providing a dual blood supply. Additionally, the lateral rami of the lateral striate arteries contribute to the blood supply, resulting in a triple vascular source. The terminal arteriolar territories of these three sources appear to interdigitate as well [107]. This pattern of vascular supply could be considered in predicting the vulnerability of various cerebral regions to anoxic or hypoperfusion states. Moody et al. [107] proposed that three key features of cerebral microvascular beds enhance the potential for collateral flow, and that the presence or absence of these features helps determine the susceptibility of a region to vascular damage:

- Continuous capillary network: In the central nervous system of placental mammals, a continuous capillary network is present, facilitating weak collateral flow between adjacent arteriolar territories.
- Multiple sources of blood supply: Some regions of the brain receive blood from two or three widely separated surface (pial) arterial sources, providing a more robust collateral supply.
- Interdigitation: This refers to the overlapping and interpenetrating territories of adjacent arterioles. Instead of having smooth, distinct boundaries, the perfusion territories in the capillary bed fit together like a jigsaw puzzle.

The first feature, i.e., a continuous capillary network, is found throughout human white and gray matter. However, the second and third features—multiple supply and interdigitation—are typically present in specific regions, providing additional protection against vascular insults. These features often occur together and appear to confer an increased collateral flow.

The regions of the human cerebrum with interdigitating arterioles from different parent arteries include subcortical U fibers and the external capsule–claustrum–extreme capsule area. These regions have a higher likelihood of collateral blood supply if surface arteries become narrowed, as interdigitated arterioles can compensate by drawing from separate, undiseased surface sources. In contrast, other brain regions with non-interdigitating arterioles have more isolated perfusion territories. Our study found no lacunar infarcts in the U-fiber region, suggesting that interdigitation offers significant protection from ischemic events. This mechanism is particularly beneficial during hypotensive events or when vascular conditions are already compromised.

In the cerebrum, areas with interdigitating arterioles tend to also have dual or triple blood supplies, although this is not always the case. The pia-arachnoid lacks capillaries, and the brain’s surface arteries form a connected plexus. While regions like the cortex and corpus callosum are not interdigitated, they still have some protection from hypotension due to the proximity of the pial plexus and their afferent supply from arterioles, which are less prone to atherosclerosis.

On the other hand, regions such as the centrum semiovale, basal ganglia, and thalamus, which lack interdigitating arteriolar fields and are supplied by long arteries, are particularly vulnerable to ischemic events. These areas are more likely to suffer from small ischemic (lacunar) infarcts due to the susceptibility of their arteries to narrowing from atherosclerosis, especially in conditions like hypertension and diabetes. Over time, these arteries can develop deformations such as twists, spirals, and loops, which further reduce perfusion by increasing the vessel length and altering the flow direction, ultimately making these regions more prone to ischemia.

The umbrella of vascular parkinsonism (VaP) contains a plurality of clinical and neuroimaging subcategories, well summarized by Viczarra et al. [108]. A thorough review of the literature reveals several key findings regarding VaP. First, no distinct structural imaging pattern is uniquely associated with VaP. Second, isolated white matter hyperintensities on brain MRI show a limited correlation with small-vessel disease (SVD)-related parkinsonism, as evidenced by the available clinico-pathologic data. Third, true parkinsonism caused by vascular injury (“definite” vascular parkinsonism) typically arises from ischemic or hemorrhagic strokes affecting the substantia nigra (SN) and/or the nigrostriatal pathway, while sparing the striatum, cortex, and connecting white matter (WM). The other clinical categories to deal with are pseudovascular parkinsonism (parkinsonism accompanied by nonspecific neuroimaging abnormalities), vascular pseudoparkinsonism (e.g., akinetic mutism due to bilateral mesial frontal strokes or apathetic depression from bilateral striatal lacunar strokes), and pseudovascular pseudoparkinsonism (higher-level gait disorders—HLGDs—including normal pressure hydrocephalus—NPH). The main limitation in this field is the lack of pathological validations, but in PD, the role of the claustrum received pathological support, and a hypothesis of its involvement in vascular parkinsonism, with or without direct injury to the claustrum, is plausible. From a pathological point of view, an interesting aspect is provided by the ex vivo 7T MRI studies with documentation of iron deposits in multiple brain structures, including the claustrum, in patients with neurodegenerative and cerebrovascular diseases [109]. In this study, the regions of interest were the hippocampus, claustrum, caudate nucleus, putamen, globus pallidus, thalamus, mamillary body, lateral geniculate body, subthalamic nucleus, red nucleus, substantia nigra, and dentate nucleus of the cerebellum. The claustrum was the main site for iron deposits in neurodegenerative and overlapping neurodegenerative–vascular diseases, in particular, fronto-temporal lobar degeneration (FTD). Claustrum lesions are consistently observed in nearly all cases of PD [85]. Cognitive complications associated with PD, including dementia, functional decline, and behavioral impairments, are thought to be mediated by claustral dysfunction. Cognitive impairment in Parkinson’s patients is linked to damage in the white matter of the telencephalon, and the claustrum, with its extensive connections, is believed to play a significant role in this decline [55,110]. In PD, there is a notable reduction in claustral connectivity with the cortical regions involved in visual–motor and auditory processing. Specifically, a decreased connectivity is observed with the parietal cortex, upper temporal and postcentral regions, middle temporal gyrus, and areas of the frontal gyrus, including the pars opercularis, pars triangularis, and pars orbitalis [111]. Functional and anatomical disorders of the claustrum are thus strongly associated with PDD [111]. Atrophic lesions in the claustrum may reflect underlying pathophysiological changes in PD [20,112]. Additionally, PD patients exhibit significantly reduced levels of dopamine and norepinephrine in the claustrum [112]. This deficiency is believed to disrupt mechanisms of information processing, further contributing to the cognitive and functional impairments observed in PD.

**Figure 5:**
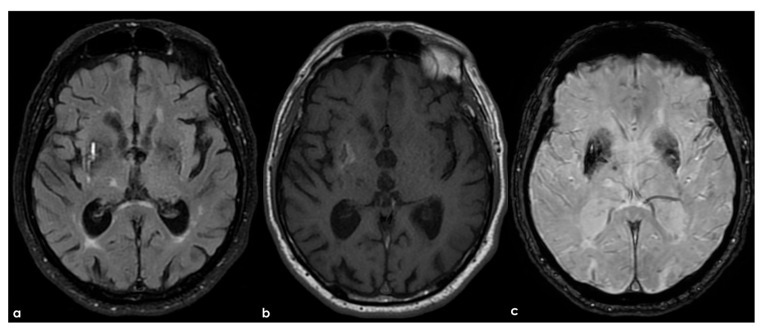
Brain MRI of a patient with an acute onset of a gait disorder and left hemisensory syndrome. The brain MRI was performed 2 months after symptom onset and the neurological examination found lower body parkinsonism. Panel (**a**) shows the axial fluid attenuated inversion recovery (FLAIR) sequence, panel (**b**) shows an axial T1W sequence, and panel (**c**) shows an axial susceptibility weighted sequence. A right (FLAIR hyperintense and T1W hypointense) hemorrhage occurred involving the lateral aspect of the putamen and the claustrum.

Then, two different scenarios are possible and deserve consideration: the sudden occurrence of an extrapyramidal syndrome due to an acute stroke involving the claustrum, directly or indirectly, and the claustrum’s role in chronic extrapyramidal syndrome within SVD. The first scenario relies on isolated reports. One of them [113] described a case of subacute-onset gait abnormalities and cognitive dysfunction without specific features, initially attributed to NPH because of the simultaneous presence of ventriculomegaly. The authors postulated that a right-sided infarction of the putamen and claustrum might explain the symptoms, in particular because of the sudden onset with the left hemiparesis, followed by a significant change in gait and cognition. The neurological examination was supportive of lower body predominant parkinsonism and the gait disorder was interpreted within the category of HLGDs, as vascular parkinsonism with a unilateral infarction in the right claustrum and putamen [108,114]. Another report [115] proposed a case of an acute stroke involving the left claustrum and provoking an ataxic gait. An example of this scenario is illustrated in Figure 5.

**Figure 6:**
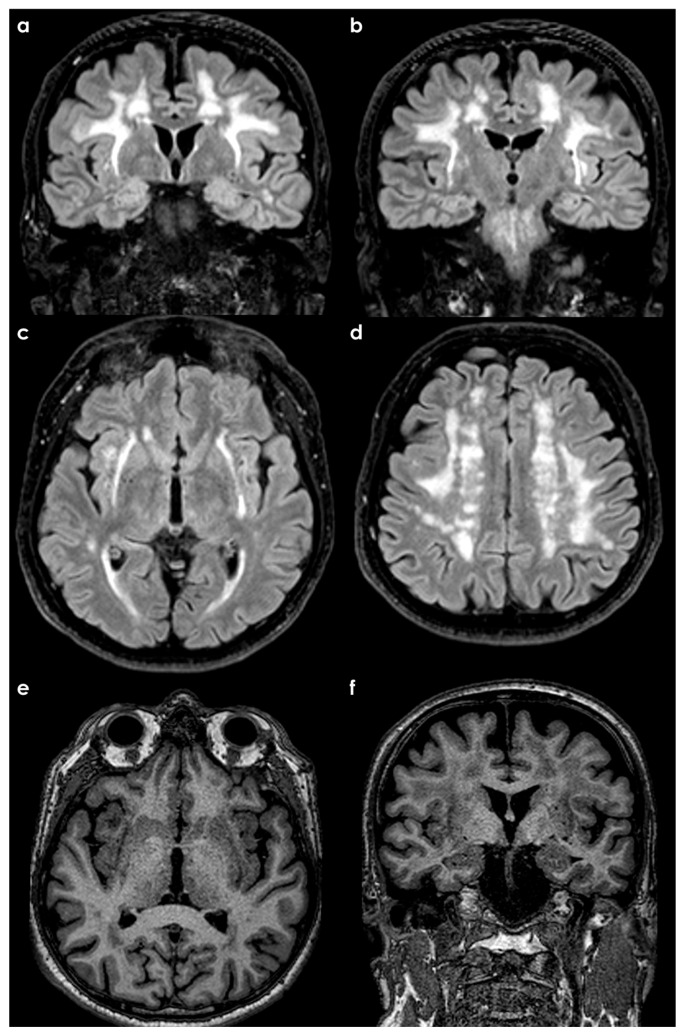
Brain MRI of a patient with extensive SVD and mild lower body parkinsonism with memory complaints. Panels (**a**–**d**) show FLAIR (coronal in (**a**,**b**), axial in (**c**,**d**)) sequences with extensive white matter hyperintensities extending along the external capsule on both sides and in the extreme capsula on the right side. Panels (**e**,**f**) show the corresponding axial and coronal T1W sequences with a hypointense and easily identifiable claustrum on both sides.

Research on the functional connectivity of the human claustrum has highlighted its asymmetric nature. Specifically, the right claustrum exhibits significant functional connectivity with the fronto-parietal and dorsal attention networks. These findings provide compelling evidence that the claustrum plays a pivotal role in coupling with the fronto-parietal network, collaboratively facilitating the initiation of new task states. This is achieved through its ability to flexibly modulate and interact with other control and processing networks [116,117]. Furthermore, claustral asymmetry was reported not only in the literature related to neurological disorders [19,59,85,118,119,120], but also related to cognitive processes [121,122,123]. The second scenario has been even less addressed in the literature, and an example is provided in Figure 6.

In a prospective study about the relation between SVD and gait, one of the most interesting issues is that the areas with the highest infarct probability were the bilateral frontal subcortical white matter and deep gray structures (claustrum, putamen) and the insula [124], but this issue was not deeply analyzed.

## 7. Insights and Prospects for the Claustrum as a Therapeutic Target in Parkinsonism

The claustrum is a small, yet complex, brain structure with extensive connections to clinically relevant regions such as the substantia nigra, striatum, and cortical motor areas. Its role remains enigmatic, but it is thought to span motor, perceptual, and cognitive domains. These functions, often disrupted in PD, suggest a potential involvement of the claustrum in such disorders. Despite its significance, the claustrum’s small size and proximity to the striatum have made its investigation challenging through imaging techniques. Emerging evidence highlights the claustrum’s relevance in neurodegenerative conditions. Early pathology in PD reveals dopamine and noradrenaline depletion in this region, while other syndromes, such as the MSA-parkinsonian variant (MSA-P) and progressive supranuclear palsy (PSP), show claustral atrophy. Furthermore, the claustrum exhibits distinct imaging features in Wilson’s disease.

Recent studies using lesion network mapping [125,126,127] and deep brain stimulation (DBS) [128,129,130,131,132,133,134] have advanced our understanding of the claustrum’s role. Connectivity analyses show that lesion-induced parkinsonism overlaps with neurodegenerative patterns in conditions like PD, PSP, and MSA-P. Importantly, DBS targeting the subthalamic nucleus (STN) connects to claustral networks, correlating with clinical improvement. These findings suggest that the claustrum is a potential therapeutic node, where various parkinsonian syndromes may converge [135,136].

While traditional DBS targets like the STN are effective for idiopathic PD, they may not address other forms of parkinsonism linked to different neural circuits. This underscores the need for the further exploration of the claustrum as a target for tailored interventions. Future research integrating functional and structural connectivity approaches could refine strategies for neuromodulation, expanding therapeutic options across diverse parkinsonian disorders.

## 8. Conclusions

The role of the claustrum in extrapyramidal networks is still a matter of debate in health and disease. Several pathological and functional studies have identified a high rate of claustrum abnormalities in PD, and a crucial role of the claustrum in the non-motor symptoms of PD has been postulated. Scarce data exist in vascular parkinsonism, but the hypothesis of a claustrum involvement is plausible. Nevertheless, this needs to be better detailed in dedicated prospective studies.

## Figures and Tables

**Table 1:** Anatomy of the human claustrum.

Segment	Features
Dorsal Claustrum (Insular Claustrum)	A continuous, irregular lamina of gray matter situated between the putamen (separated by the external capsule) and the insular cortex (separated by the extreme capsule). It has a triangular cross-sectional shape, narrowing superiorly and widening inferiorly. The external capsule, in contrast, widens superiorly and becomes thinner or absent near the lower dorsal claustrum.
Ventral Claustrum (Fragmented Claustrum)	It is composed of diffuse, island-like gray matter fragmented by the uncinate and inferior occipitofrontal fascicles. It has superior and inferior parts: The superior ventral claustrum connects to the anteroinferior pole of the dorsal claustrum and extends toward the base of the frontal lobe near the prepiriform cortex. The inferior ventral claustrum connects to the posteroinferior pole of the dorsal claustrum and extends toward the amygdalar region, with which it shares a close anatomical relationship, and it is often difficult to delineate.

**Table 2:** Main pathological changes in the claustrum in PD patients.

Pathological Changes	Details
Lewy Body (LB) and Lewy Neurite (LN) Pathology	- Presence of LBs and LNs in the claustrum, with LB morphology resembling cortical-type inclusions (less distinct than brainstem LBs). - LNs exhibit varied morphologies, including serpentine and pearl-like structures. - α-synuclein (αSyn) immunoreactive inclusions, including extracellular deposits, identified in PD claustrum.
Astrocytic αSyn Inclusions	- Found in the claustrum of PD patients, but not in controls, indicating a unique astrocytic pathology.
Proteinopathies	- Minimal tau pathology (neurofibrillary tangles and neuropil threads). - Amyloid-β (Aβ) deposits present in 25% of PD, 58% of PDD, and 100% of DLB cases. - Co-occurrence of αSyn, Aβ, and tau in some cases highlights shared molecular mechanisms.
Functional Connectivity Changes	- Altered connections of the left claustrum with the sensorimotor and cingulate cortex in PD, despite no significant volume differences compared to controls.
Clinical Correlations	- Dementia develops in 48–78% of PD cases, and visual hallucinations in up to 60% of cases. - Pathology in visuo-claustral pathways linked to visual hallucinations. - Implicated in cognitive decline, particularly in PDD and DLB. - Claustrum dysfunction may exacerbate disruptions in cortical–subcortical networks.
Behavioral and Psychiatric Symptoms	- Impulse-control disorders (e.g., gambling, hypersexuality) linked to disinhibition from claustral changes. - Sleep disturbances potentially due to disrupted arousal–sensory integration from claustral dysfunction. - Depression linked to reduced metabolism and dysfunction in the claustrum and associated regions.

## Data Availability

Not applicable.
